# Raman relaxation in Yb(iii) molecular qubits: non-trivial correlations between spin–phonon coupling and molecular structure

**DOI:** 10.1039/d6sc02836e

**Published:** 2026-09-14

**Authors:** Giacomo Sansone, Lorenzo A. Mariano, Stefano Carretta, Paolo Santini, Alessandro Lunghi

**Affiliations:** a Dipartimento di Scienze Matematiche, Fisiche e Informatiche, Università di Parma I-43124 Parma Italy; b INFN – Sezione di Milano-Bicocca, Gruppo Collegato di Parma 43124 Parma Italy; c School of Physics, AMBER and CRANN Institute, Trinity College Dublin 2 Ireland lunghia@tcd.ie; d Consorzio Interuniversitario Nazionale per la Scienza e Tecnologia dei Materiali (INSTM) I-50121 Firenze Italy

## Abstract

The coordination complexes of Yb(iii) exhibit some of the longest spin coherence times among 4f compounds, making them a promising platform for molecular quantum technologies. While spin–phonon relaxation remains a limiting factor for coherence times even at low temperature, its control through chemical design has the potential to push these spin qubit prototypes beyond current limits. With the aim of providing insights into how to chemically control spin–phonon relaxation, we here present a full *ab initio* study of spin–phonon dynamics for three Yb(iii) molecules exhibiting minimal chemical differences, yet quantitatively different spin relaxation times. The results show that low-temperature relaxation is governed by Raman processes triggered by a small group of largely delocalized low-energy phonons. The analysis of these contributions highlights that the modulation of spin–phonon coupling by molecular structure modifications beyond the first coordination shell is highly non-trivial in nature and hard to rationalize in simple chemical terms. These findings call for a conceptual step change from the attempt to use simple magneto-structural correlations to interpret the effect of molecular structural modifications on spin–phonon relaxation and present predictive first-principles frameworks as a potential driving force of future chemical design strategies.

## Abstract 2

*Ab initio* simulations of nearly isostructural molecular qubits show that low-*T*relaxation is triggered by delocalized phonons. Small chemical modifications produce non-trivial modulations of spin–phonon coupling, challenging standard design rules.

## Graphical abstract

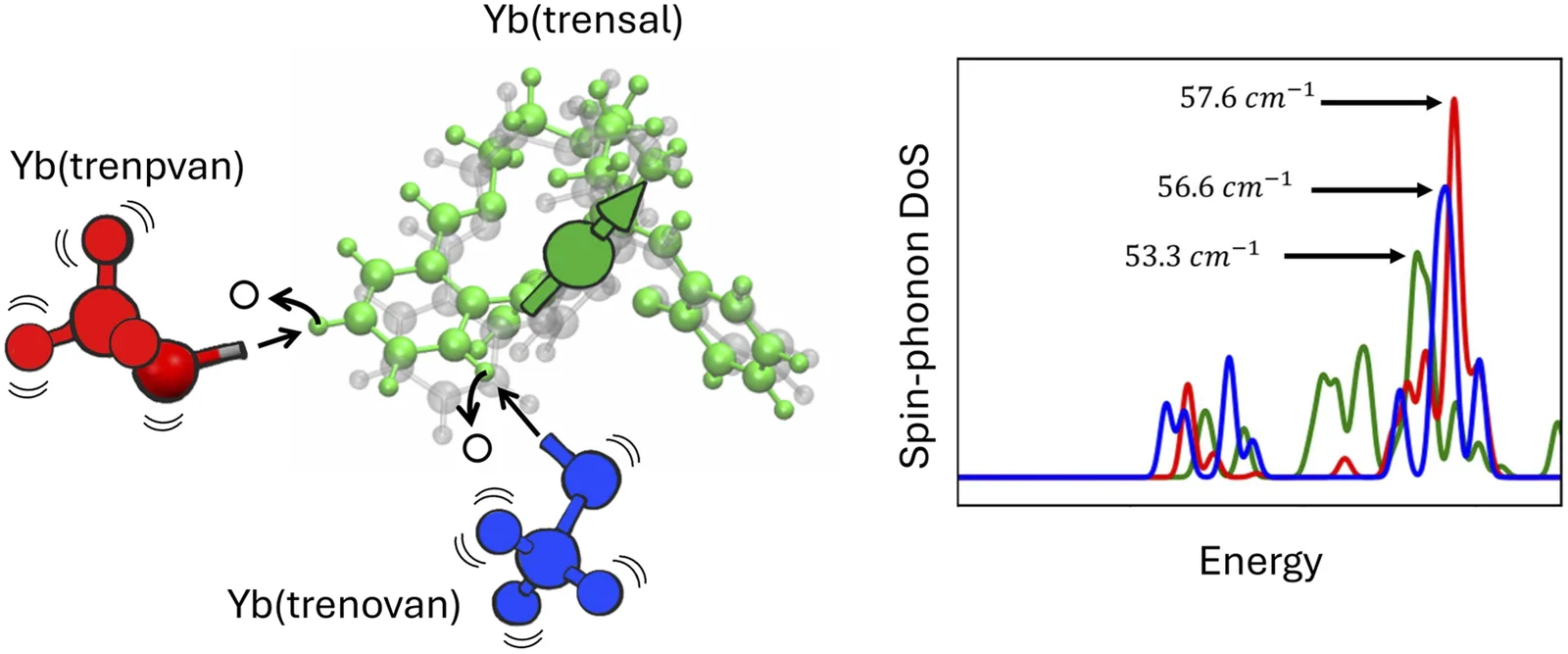

## Introduction

Lanthanide (Ln)-based 4f Molecular Nanomagnets (MNM) have been recently proposed as physical architectures for quantum information technologies due to their long relaxation and coherence times.^1–4^ A prototypical example is represented by the Yb(trensal) (1) molecule,^5^ whose ^173^Yb isotope has been vastly investigated and characterized over the last few years. High resolution spectroscopies have revealed that within the ^2^F_7/2_ ground term, the first excited electronic doublet lies nearly 500 cm^−1^ above the ground doublet.^5,6^ This allows treating Yb(trensal) as an effective electronic spin qubit (*S* = 1/2) coupled through hyperfine interaction to a nuclear spin (*I* = 5/2). With 12 addressable spin states, the Hilbert space can be expanded by encoding quantum information in a multi-level system (qudit),^7–10^ with the advantage of reducing the number of distinct physical units and two-body gates required for the implementation of many quantum operations. Transient nutation experiments with pulsed electron paramagnetic resonance (EPR)^1^ and nuclear magnetic resonance (NMR)^11^ have demonstrated the possibility to generate and coherently manipulate pair superpositions of electro-nuclear states. In addition, magnetically diluted single crystals of Yb(trensal) have been placed on the inductors of lumped-element LC superconducting resonators, achieving high cooperativity coupling to all electronic and most nuclear spin transitions, paving the way to the realization of qudit protocols in hybrid molecular spin–microwave photon processors.^12^ Moreover, a recent study has reported the realization of the first prototype quantum simulator based on an ensemble of molecular qudits, exploiting as quantum hardware a crystal containing isotopically enriched ^173^Yb(trensal), doped at 1% into its diamagnetic [Lu(trensal)] isostructural analogue, and a radiofrequency broadband spectrometer.^13^ A quantum Fourier transform algorithm could also be implemented, as recently shown in ref. 14.

Despite all this progress, the interactions of the spin degrees of freedom with the external environment lead to fast decoherence above 10–20 K,^1^ which limits the temporal window within which it is possible to coherently control the spin states.^15^ Above cryogenic temperatures (*T* > 4 *K*), the main limiting factor for coherence is the spin–phonon coupling, which sets the relaxation time *T*_1_. In Yb(trensal), this mechanism completely suppresses coherent dynamics already above a few tens of Kelvin. Understanding how structural changes in molecular crystals affect spin dynamics is essential for the rational design of molecules with tailored properties. Substantial structural modifications that strongly perturb the spin states, the phonon spectrum, or both, are difficult to rationalize, as they complicate the disentanglement of the underlying mechanisms. A more effective starting point is to implement minimal structural modifications and systematically quantify their effects on the static and dynamic properties of the molecule. In this regard, one of the first fully experimental seminal studies focused on the identification of the role of structural changes in the spin-relaxation in potential molecular quantum bits was performed on four vanadium(iv)-based systems.^16^ More recently, this idea has been applied precisely to Yb(trensal).^17^ Two new derivative complexes, namely Yb(trenpvan) (2) and Yb(trenovan) (3), were indeed synthesized by modifying the trensal backbone. In particular, in each of the ligands constituting the tripodal scaffold, one hydrogen atom is substituted with a methoxy group (R–O–CH_3_). Note that these compounds have identical chemical formulas and differ only in the placement of the methoxy group on the ligands, being either *ortho*- (Yb(trenovan)) or *para*- (Yb(trenpvan)) to the phenoxo group. Moreover, they crystallize in the same trigonal space group, namely *P*3̄, and they are characterized by very similar unit cell parameters.^17^Fig. 1 shows the structure of 1 along with those of its two derivatives, 2 and 3. A multi-technique study of the crystal field and spin–lattice relaxation of the two systems in the bulk phase was recently performed.^17^ That study revealed clear differences in the relaxation dynamics of these Yb coordination compounds despite their nearly identical spin states, thus indicating that even minor modifications of chemical groups in the second coordination sphere can affect the phonon spectrum and spin–phonon coupling. In the same work, infrared spectroscopy measurements also revealed differences in the optical phonon modes, suggesting them to be a possible origin of the observed diversity in relaxation behavior. However, a clear understanding of the relaxation mechanism in Yb^3+^ coordination complexes is still lacking, making it so far impossible to establish a direct link between chemical structure modifications and spin–phonon relaxation.

**Fig. 1:**
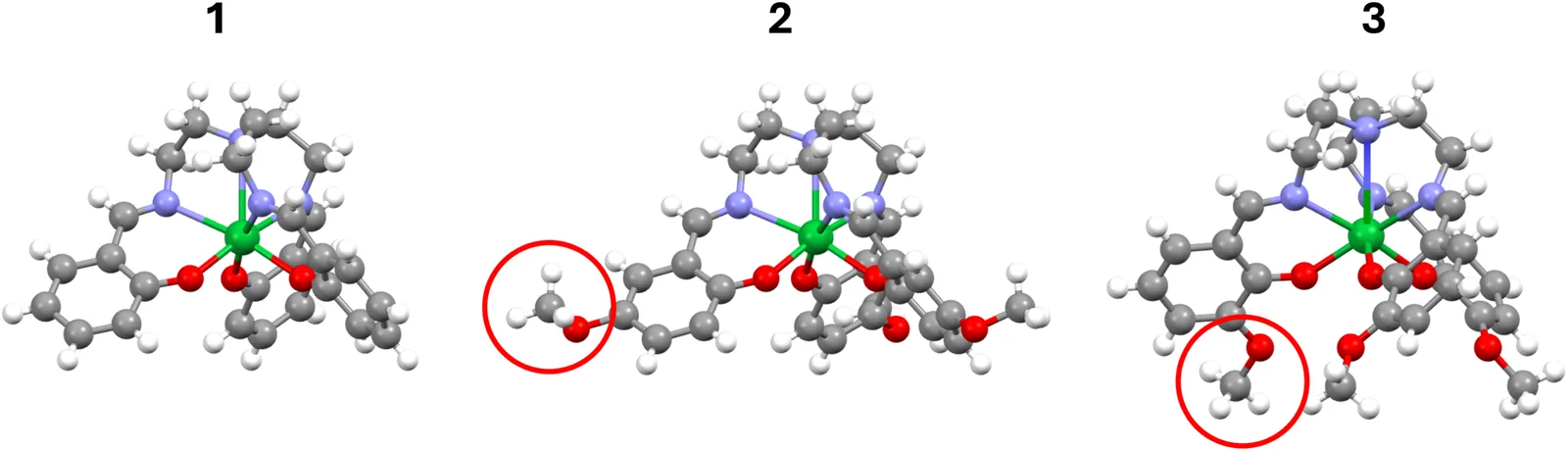
The molecular structure of Yb(trensal) (1), Yb(trenpvan) (2) and Yb(trenovan) (3). Color code: Yb = green, N = purple, O = red, C = grey, H = white. The red circles highlight the position of the methoxy groups that differentiate the three molecules.

The picture that emerges from these studies underscores the need for a deeper understanding of the interplay between structural and electronic properties and relaxation dynamics. In this context, first-principles approaches to spin–phonon relaxation provide a natural and powerful framework to address this problem.^18^ Despite the growing interest in Yb(iii) molecular qudits, a first-principles description of their spin–phonon relaxation is still lacking. Here, we address this issue through a fully *ab initio* study of spin–phonon relaxation in the family of nearly isostructural Yb(iii) molecules introduced above, demonstrating how minimal chemical modifications can subtly tune Raman relaxation pathways. The article is organized as follows: first, we report the fundamental theory behind spin–phonon relaxation processes. Then, we illustrate and discuss the results of our simulations, both for the static properties and the relaxation dynamics of this family of qubits. Finally, we report the details of the numerical methods employed in the *ab initio* calculations of phonons, spin–phonon coupling and relaxation dynamics.

## Theoretical background

The problem of decoherence induced by spin–phonon coupling in the family of Yb(tren-) molecular qubits can be modelled through the following Hamiltonian:

1 *Ĥ* = *Ĥ*_s_ + *Ĥ*_ph_ + *Ĥ*_sph_.

Here, *Ĥ*_s_ is the Hamiltonian describing the central system made of the ^173^Yb^3+^ ion, *Ĥ*_ph_ captures the contributions from the intrinsic degrees of freedom of the phonon bath, whereas *Ĥ*_sph_ models the spin–phonon coupling. The central system Hamiltonian has the form2
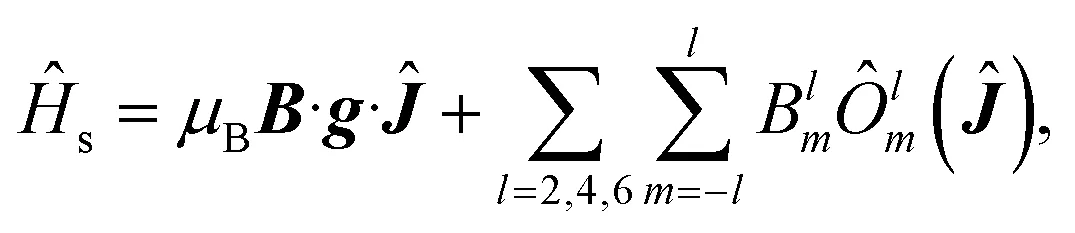
where the first term describes the interaction between the ion spin ***Ĵ*** and the external magnetic field ***B*** (with *µ*_B_ and ***g*** denoting the Bohr's magneton and the Landé tensor, respectively), while the second term describes the effective spin Hamiltonian, here modeled as a series of tesseral operators of order *l* and component *m*, *Ô*^*l*^_*m*_, and the related coefficients *B*^*l*^_*m*_. The phonon bath Hamiltonian is represented by the sum of quantum harmonic oscillators,3
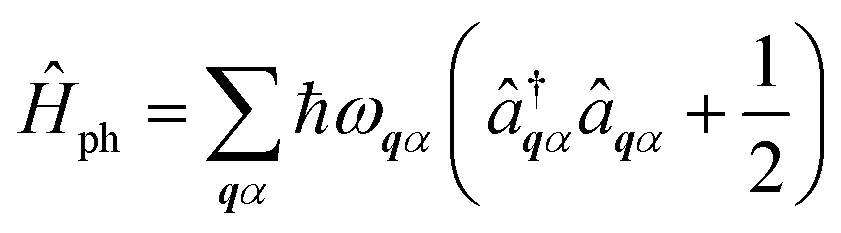
Here, ℏ is the reduced Planck constant, while *ω*_***q****α*_ is the frequency of the phonon mode *Q*_***q****α*_ in branch *α* with reciprocal lattice vector ***q***. Finally, *â*^†^_***q****α*_ and *â*_***q****α*_ are the phonon-specific creation and annihilation operators, respectively. From now on, we will drop the subscript ***q*** since our analysis will pertain only phonon modes at the *Γ*-point, *i.e.****q*** ≡ (0, 0, 0). We note that while the full Brillouin zone integration is in principle needed, previous studies have shown that it has negligible effects on the computed *T*_1_.^19^ Given the steep computational cost of such a calculation, we here only consider the Gamma point. Further details that provide a justification to this choice can be found in the SI, Section I.

The spin–phonon coupling Hamiltonian *Ĥ*_sph_ allows us to describe the modulation effect of the lattice dynamics on the spin Hamiltonian coefficients, *i.e.* the parameters *B*^*l*^_*m*_ in eqn (2). Under the assumption of a weak coupling regime between the electronic spin and phonons, we can expand the spin Hamiltonian parameters in a Taylor series with respect to the set of vibrational mode coordinates, {*Q*_*α*_}, around the equilibrium position {*Q*_*α*_ = 0},4
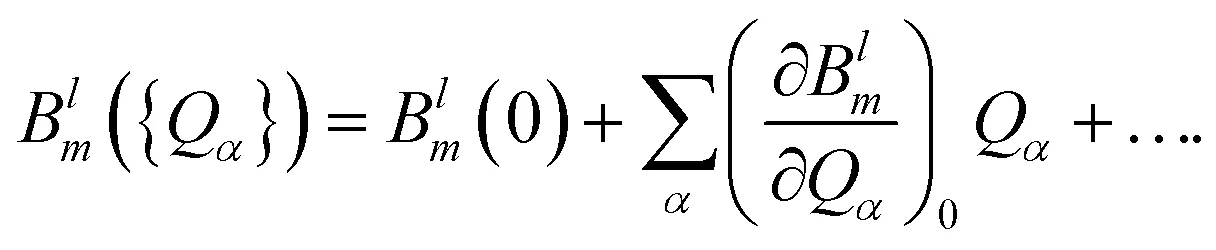
Here, *B*^*l*^_*m*_(0) represents the equilibrium spin Hamiltonian parameters appearing in eqn (2), while the first derivatives are the linear spin–phonon coupling coefficients. The subscript 0 indicates that these derivatives are evaluated at the crystal equilibrium positions. By promoting the coordinates *Q*_*α*_ to quantum operators *Q̂*_*α*_ we can then define the spin–phonon coupling Hamiltonian as5
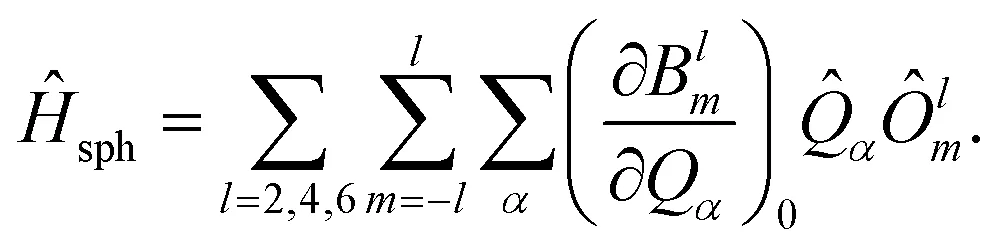


Once the eigenstates |*a*〉, |*b*〉, … of the spin Hamiltonian and the corresponding eigenvalues *E*_*a*_, *E*_*b*_, … have been obtained, it is possible to evaluate the transition rates between different spin states, *R*_*ba*_, and use them to simulate the spin dynamics through time-local quantum master equations. In the following, we will consider contributions to relaxation coming from both one- and two-phonon processes. Starting from one-phonon processes, the transition rate among two generic spin states, *R*^1-ph^_*ba*_, reads6
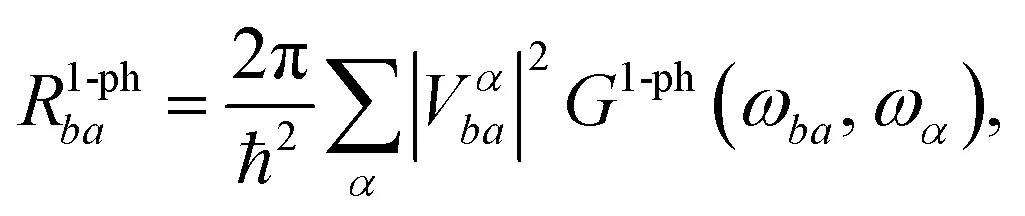
where7
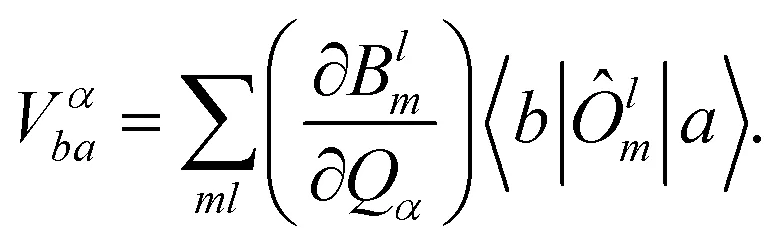
Here, the term (∂*B*^*l*^_*m*_/∂*Q*_*α*_) quantifies the intensity of the coupling between the spin and the phonon mode *Q*_*α*_, whereas ℏ*ω*_*ba*_ = *E*_*b*_ − *E*_*a*_ defines the energy gap between the two states involved in the spin transition. The function *G*^1−ph^ arises from the evaluation of the matrix element of the normal mode operator *Q*_*α*_ between two phonon eigenstates and reads

8 *G*^1-ph^(*ω*, *ω*_*α*_) = *δ*(*ω* − *ω*_*α*_)*n̄*_*α*_ + *δ*(*ω* + *ω*_*α*_)(*n̄*_*α*_ + 1),

where *δ*(*ω* ± *ω*_*α*_) is the Dirac's delta function that selects phonons resonant with the spin gap in the absorption and emission processes, and *n̄*_*α*_ = (e^ℏ*ω*_*α*_/*k*_B_*T*^ − 1) − 1 is the Bose–Einstein distribution accounting for the phonon thermal population, with *k*_B_ being the Boltzmann constant. Two-phonon processes provide an alternative path to relaxation. For instance, the simultaneous absorption and emission of two phonons can be written as follows:9
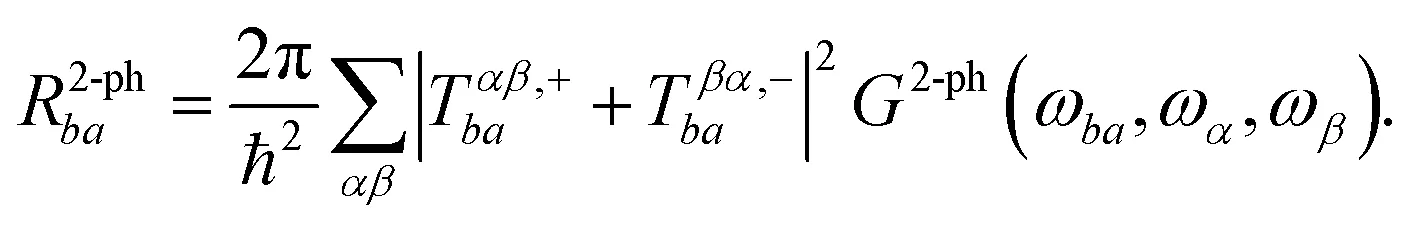
Here, the terms *T*^*αβ*,±^_*ba*_ are defined as follows:10
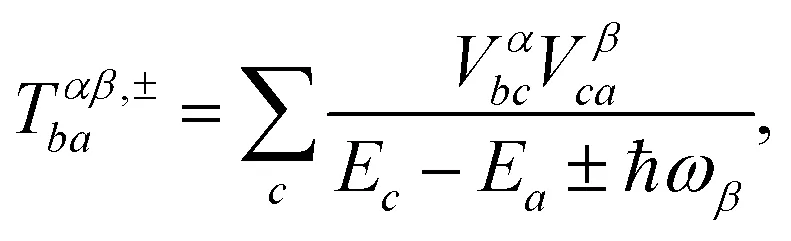
whereas the function *G*^2-ph^ acts in a similar way to *G*^1-ph^ introduced in eqn (8) for one-phonon processes, imposing energy conservation during a spin transition, and accounting for the thermal population of the states (Bose–Einstein distribution):

11 *G*^2-ph^(*ω*, *ω*_*α*_, *ω*_*β*_) = *δ*(*ω* + *ω*_*α*_ − *ω*_*β*_)(*n̄*_*α*_ + 1)*n̄*_*β*_

These two processes have been shown to be able to induce spin relaxation in coordination complexes of f-ions through Orbach and Raman-I relaxation mechanisms, respectively.^20–23^ We note that we do not consider here a second source of two-phonon processes, which originates from the second-order term in the expansion of the crystal-field parameters (eqn (4)) and leads to what is sometimes referred to as Raman-II relaxation.^23^ These contributions would lead to a spin relaxation mechanism that, like the direct one, is suppressed by the Kramers nature of the Yb ground-state doublet. This is little affected by the applied magnetic field, which is too small to induce appreciable mixing with excited states given their large excitation gaps shown in Fig. 2.

**Fig. 2:**
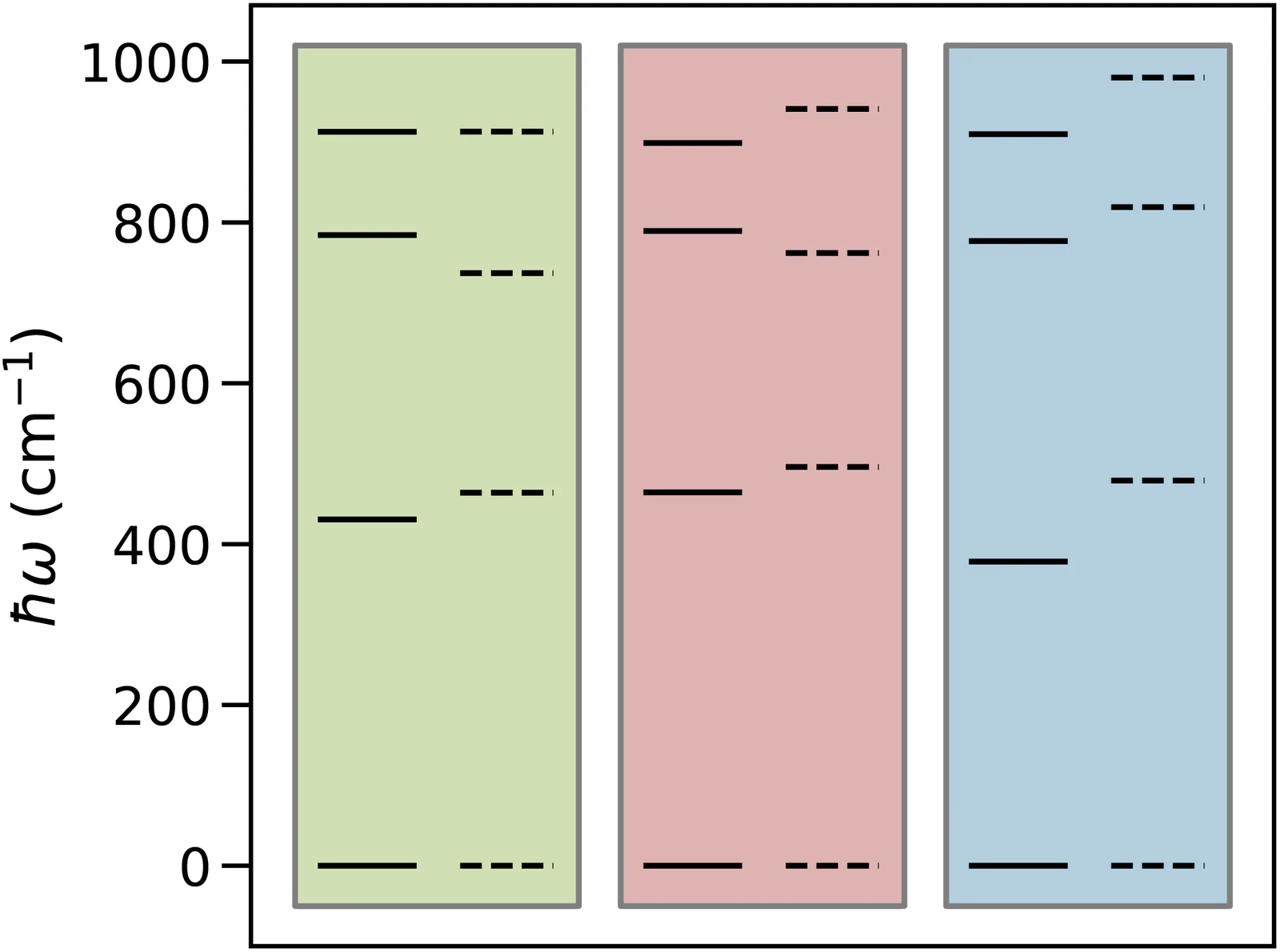
Energy spectra of the ground term ^2^F_7/2_ for the three systems. The solid lines represent the energy levels retrieved from NEVPT2 calculations, whereas the dashed lines refer to the experimental spectroscopic values.^5,6,17^

The relaxation rates computed through (6) and (9) allow us to build a transition rate matrix *R*_*ba*_, which contains the whole information related to the dynamics of the population (diagonal) terms of the density matrix, namely12
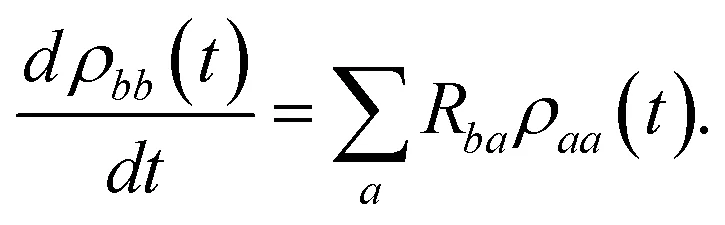


A generalization of these equations to the full density matrix, thus including the effect of phonons on coherence terms, has recently been proposed,^24^ but here we will only be concerned with the study of population terms, which are decoupled from coherences in the absence of spectral degeneracies.^23^

## Results

Before discussing our results, it is useful to recall why Ln(iii)-based molecular qubits typically display faster spin relaxation than transition-metal-based systems such as V(iv).^25^ In 3d ions, the crystal-field splitting largely dominates over the spin–orbit coupling. This leads to a quenching of the orbital angular momentum and yields a nearly isotropic, spin-only *g*-tensor weakly coupled to lattice vibrations. On the other hand, in 4f ions such as Yb(iii), the opposite hierarchy of interactions holds: spin–orbit coupling dominates over the crystal field, so the total angular momentum *J* remains a good quantum number within the spectroscopic term. This results in the large, anisotropic *g*-tensor reported in Table 1 and is directly responsible for the stronger spin–phonon coupling, and therefore for faster Raman/Orbach relaxation, observed in Ln(iii) qubits relative to their transition-metal counterparts. The larger magnetic moment associated with this anisotropy also enhances electronic dipole–dipole interactions between neighbouring spin centers, making Ln(iii) systems considerably more sensitive to sample concentration; this is why magnetically diluted samples (*e.g.*, 1% doping into a diamagnetic isostructural host, as used for the experimental data compared here) are required to approach the intrinsic, dipole-free relaxation regime targeted by our simulations.^4^

**Table 1:** Comparison between simulated and experimental *g*-tensor values for the ground doublet of the three systems

Molecule	Method	*g*_*xx*_	*g*_*yy*_	*g*_*zz*_
Yb(trensal)	Sim.	2.68	2.73	4.88
Yb(trensal)	Exp.	2.9	2.9	4.3
Yb(trenpvan)	Sim.	2.51	2.90	4.91
Yb(trenpvan)	Exp.	2.96	2.96	4.16
Yb(trenovan)	Sim.	2.46	2.61	5.24
Yb(trenovan)	Exp.	2.65	2.65	4.89

The energy spectra related to the spectroscopic ground term of Yb^3+^, ^2^F_7/2_, consist of four Kramers Doublets (KDs). The results of their *ab initio* calculation are summarized and compared to experimental measurements^5,6,17^ in Fig. 2. We observe excellent agreement between the calculated and experimental values, with a maximum deviation of ∼10% for the first excited doublet of Yb(trenovan). Table 1 summarizes the results related to the *g*-tensor for the ground state doublet. With respect to the experimental values, the theoretical results tend to overestimate the *g*-shift. This is a feature that has been already observed in previous studies on Yb^3+^ specifically and on Ln-based complexes in general.^6,26^ While these discrepancies in the *g*-tensor components reflect small inaccuracies in the Kramers doublet wavefunctions, the excellent agreement of the energy spectra (Fig. 2) ensures that the electronic structure is well described for the purposes of relaxation dynamics. Furthermore, we observe that the obtained *g*-factors are not perfectly axial (*g*_*xx*_ ≠ *g*_*yy*_), as one would expect from a system with trigonal symmetry. The reason behind this result lies in the geometry optimization procedure required to compute the vibrational properties: in order to obtain physically sound phonon frequencies, *i.e.* avoiding imaginary modes, it is necessary to find the molecular structure in a local energy minimum on the potential energy surface. The unconstrained optimization to minimize the energy of the unit cell structure through pDFT methods can introduce a symmetry-breaking distortion that affects the xy plane isotropy of the *g*-tensor. Nevertheless, since the relaxation dynamics is dominated by the spin–phonon coupling (see eqn (7) and (10)), we expect these deviations from the ideal case to have a negligible impact on the *ab initio* description of the process. An in-depth study in support of these observations is presented in Section II of the SI.

The spin–phonon relaxation time *T*_1_ was computed for the three systems as a function of temperature. The results are compared with experimental data in Fig. 3a–c. Notably, low-temperature spin relaxation is subject to multiple contributions, most importantly spin cross-relaxation effects, which are not captured by the state-of-the-art relaxation theories due to the neglect of dipolar interactions among multiple molecular units. For this reason, it is essential to compare simulations with experiments carried out at the highest possible dilution levels, and we therefore made the conscious choice of referring to the data corresponding to the most diluted samples available for all three systems. Data related to 1 were taken from Bode *et al.*,^27^ where pulsed EPR measurements were performed on a single-crystal of diamagnetic Lu(trensal) at 1% Yb doping (Lu_0.99_Yb_0.01_(trensal)), with an external field *B*_0_ = 0.2012 T oriented along the *C*_3_ crystallographic axis. Data for 2 and 3 were taken from Hansen *et al.*,^17^ and were obtained through dynamic magnetic susceptibility measurements on diluted Lu_0.99_Yb_0.01_(trenpvan) and Lu_0.99_Yb_0.01_(trenovan), under an external field *B*_0_ = 0.05 T also oriented along the *C*_3_ axis. The calculations were performed assuming an external magnetic field *B*_0_ = 0.05 T for all three systems, oriented along the *C*_3_ crystallographic axis. In the case of 1, the simulation was performed also by setting *B*_0_ = 0.2012 to perfectly match the experimental conditions. However, no appreciable dependence of *T*_1_ on the field was noticed within the range of such small magnetic fields (see Fig. S4).

**Fig. 3:**
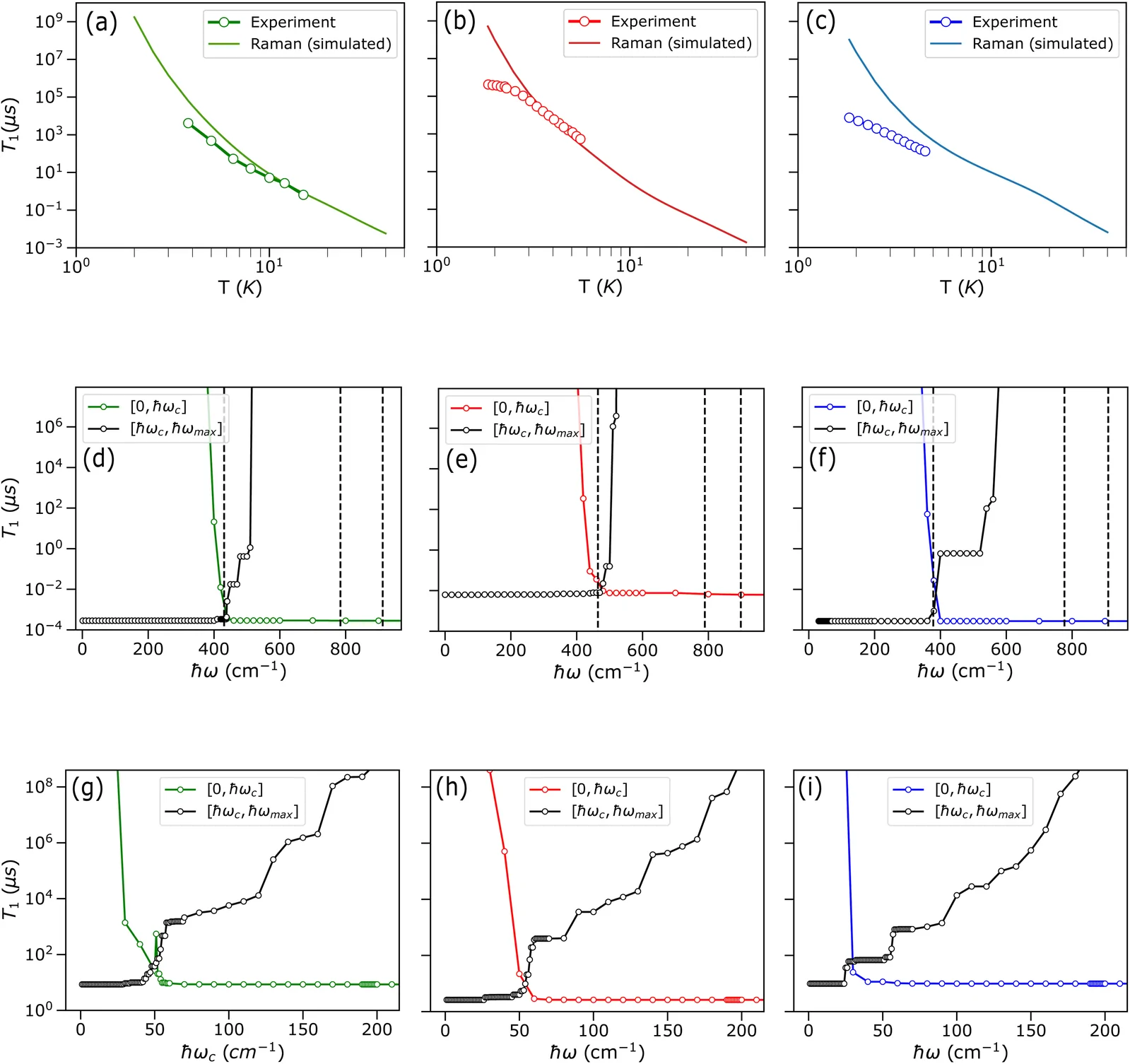
Relaxation time as a function of temperature for Yb(trensal) (a), Yb(trenpvan) (b) and Yb(trenovan) (c). Simulated Raman (solid line) and Orbach (dashed) contributions are reported. Experimental data (hollow circles) are taken from ref. 17 and 27. Panels (d)–(f) and (g)–(i) report the results of the energy cut-off analysis for each system, both in the case of Orbach relaxation (panels (d)–(f), *T* = 100 *K*) and Raman relaxation (panels (g)–(i), *T* = 10 K), respectively. Colored curves report the relaxation time *T*_1_ evaluated by considering phonons in the energy interval [0, *ω*_c_], where *ω*_c_ is the cut-off frequency. The black curves report the same simulation for phonons in the interval [*ω*_c_, *ω*_max_], where *ω*_max_ is the fixed, maximum frequency of the phonons considered. The vertical, dashed lines mark the energy levels of the KD.

As we demonstrate in Fig. S6 of the SI, Orbach relaxation is negligible at low temperatures, as expected from the large crystal-field gaps (see Fig. 2) and from its reliance on real, thermally activated transitions from the ground to excited doublets. Thus, two-phonon processes alone determine (Raman) relaxation in the experimentally accessible temperature window. For 1 and 2 the calculations are in good agreement with experimental data in the upper range of measured temperatures, whereas *T*_1_ is overestimated at lower temperatures. This is not unexpected, when spin–phonon relaxation becomes very slow different minor relaxation mechanisms emerge and eventually set *T*_1_ (*e.g.*, quantum tunneling and dipolar interactions). Unfortunately, in the case of 3, we lack information on *T*_1_ at temperatures higher than 5 K, making the simulated *T*_1_ overestimated over the whole range of the available measurements. It must be stressed that the differences in relaxation behavior among the three variants presented in Fig. 3 are entirely ascribable to the spin-lattice dynamics. We quantitatively demonstrate this point in Fig. S7, which shows that no substantial impact on the relaxation dynamics of 1 is observed by interchanging its static crystal field Hamiltonian with that of 2 or 3, in agreement with the high level of similarity among the electronic spectra in Fig. 2.

To get a deeper insight into the relaxation mechanism and identify the vibrational modes that most strongly influence the spin dynamics, we calculate *T*_1_ by selectively including phonon modes within specific energy windows. First, we set the lower bound of the energy window at 0 cm^−1^ while the upper bound *ω*_c_ is progressively increased; at each step, *T*_1_ is evaluated by including phonons with energies in the interval [0, *ω*_c_]. We refer to this procedure as high cut-off analysis. In a second approach, we fix the upper bound *ω*_max_ of the energy window, and the lower bound *ω*_c_ is progressively increased from 0 cm^−1^ up to *ω*_max_; again, at each step *T*_1_ is calculated considering the contribution of phonons with energy in the interval [*ω*_c_, *ω*_max_] (low cut-off analysis). The results are summarized in Fig. 3d–i. Starting from the Orbach mechanism, computed at 100 K for this analysis, we observe in Fig. 3d–f a sharp change in the relaxation time as the upper bound *ω*_c_ of the energy window approaches the energy gap between the ground state and the first excited KD. For 1, 2 and 3 this drop in *T*_1_ is registered around values closely matching the first KD excitation. This behaviour highlights the key role of the initial absorption of a phonon that leads to a real spin transition from the ground state to the first excited KD. Conversely, the behaviour of *T*_1_ upon increasing the lower bound *ω*_c_ of the phonon energy window reveals an approximately specular trend, with an almost complete quenching of relaxation when phonon modes with energies around the first excited KD are excluded. Turning to the Raman regime, computed at 10 K (see Fig. 3f–i), the high energy cut-off analysis shows a rapid decay of *T*_1_ upon inclusion of phonons within 30–100 cm^−1^ for all three systems, demonstrating the role of low-energy phonons in Raman relaxation. Convergence is reached at ≈70 cm^−1^ for 1, ≈90 cm^−1^ for 2 and ≈60 cm^−1^ for 3. The low-energy cut-off analysis shows that an almost complete quenching of the relaxation is reached only after the exclusion of the phonon modes up to 200 cm^−1^. This indicates that a broad set of vibrational modes could provide a contribution to the Raman relaxation process that is only slightly less important than the dominating low-energy phonons.

As a final step, in order to identify the nature of the main vibrational modes that contribute to relaxation, we examine the spin–phonon coupling density of states *D*(*ω*), which is defined as follows:13
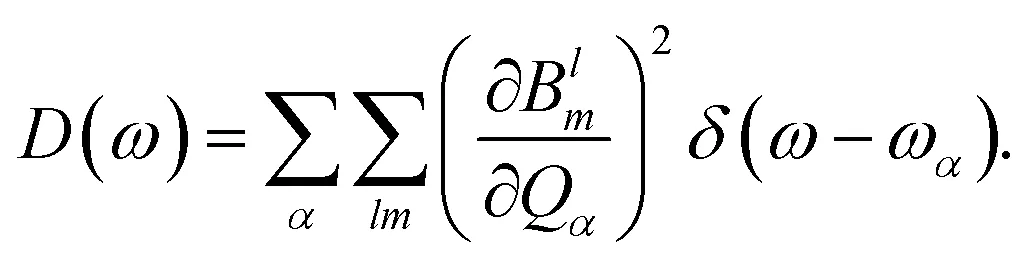


The results are reported in Fig. 4a–d, where we show both the normalized phonon density of states (black) and the quantity *D*(*ω*) rescaled with respect to its maximum value. These results show that the minor chemical differences among the three variants affect the relaxation dynamics in a subtle manner. For such localized structural modifications, phonon modes are expected to be primarily altered in the high-energy part of the spectrum (hundreds of cm^−1^), and indeed changes in this region were detected by IR spectroscopy.^17^ However, Fig. 4 reveals that the spin–phonon density of states of the three variants does not differ by a simple shift or rescaling, nor by the contribution of isolated phonon modes, but rather through an extended redistribution across the spectrum. At temperatures of the order of 10 K or below, our cut-off analysis shows that Raman relaxation is governed exclusively by phonons in the very low-energy range. The corresponding modes cannot be ascribed to the vibrations of a specific, localized group of atoms in the molecules. Indeed, a detailed inspection of the atomic displacements related to these modes clearly shows that the oscillations pertain to the molecular structure as a whole. We illustrate these findings in Fig. 4e, where we report the geometry of the molecule distorted according to the nature of low-energy phonons.

**Fig. 4:**
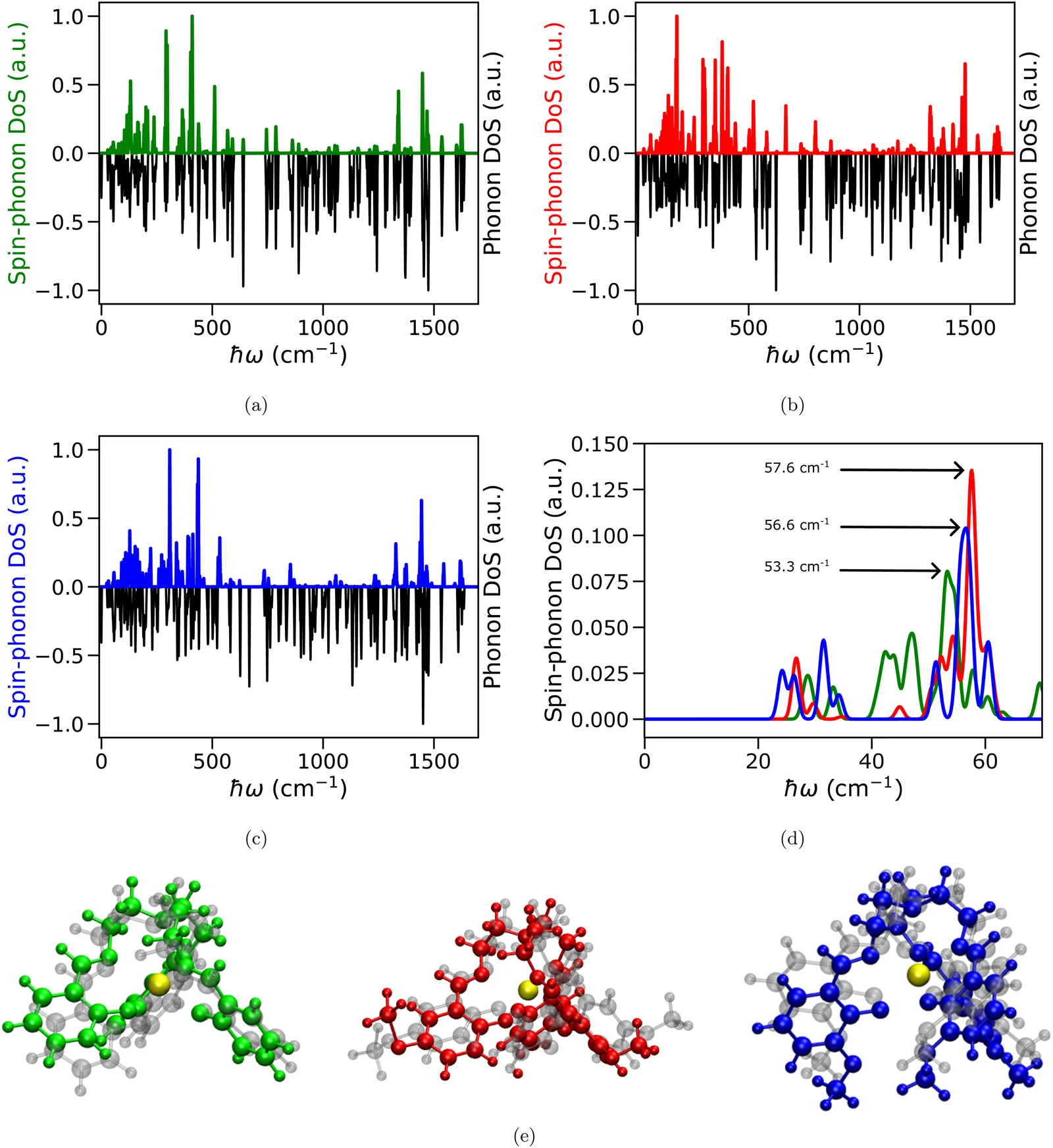
Panels (a)–(d): normalized spin–phonon density of states for Yb(trensal) (a), Yb(trenpvan) (b) and Yb(trenovan) (c). In black: normalized phonon density of states for each system. Every spectrum is rescaled with respect to its maximum value (in modulus). The scale factor is about 0.91 for Yb(trensal), 0.55 for Yb(trenpvan) and 0.75 for Yb(trenovan). (d) Zoom on the [0, 70] cm^−1^ interval. The adopted smearing is Gaussian and equal to 1 cm^−1^. Panel (e): pictorial visualization of the low-energy oscillation modes, corresponding to the highest peaks of the spin–phonon DoS reported in (d), appearing at 53.3 cm^−1^ (green), 56.6 cm^−1^ (blue), and 57.6 cm^−1^ (red), respectively. The geometry of the molecule at a certain instant (coloured molecule) is superimposed to the one at the beginning of the oscillation (shaded molecule). In this portrayal, the Yb atom colour is yellow to simplify its visualization.

## Discussion

We presented a comprehensive *ab initio* study of the relaxation behavior of a family of virtually isostructural Yb(iii) coordination compounds: Yb(trensal) and its derivatives Yb(trenpvan) and Yb(trenovan). The derivatives differ only by the substitution of a hydrogen atom with a methoxy group at distinct positions on the trensal backbone with respect to the phenoxo group: *ortho*- in the case of Yb(trenovan) and *para*- for Yb(trenpvan). This results in minimal structural perturbations, yet sizable effects on spin relaxation. Multireference electronic structure calculations, in agreement with available experimental data, reveal nearly identical electronic excitations across the series, coherent with a high degree of similarity in their first coordination shells. This exceptional degree of electronic similarity makes these compounds an ideal platform to isolate and quantify the role of phonons in spin–lattice relaxation dynamics, which, unlike crystal field splitting, is expected to be influenced by the full molecular structure, beyond the first coordination shell, and the molecular crystal packing.

As briefly mentioned in the Theoretical background section, the role of the direct mechanism is known to only take place at high fields, where the ground-state Kramers doublet is split enough for phonons with large spin–phonon coupling and density of states to play any significant role. For the small external magnetic field *B* = 0.05 T used in our simulations, the Zeeman splitting can be estimated to be Δ*E*_Zeem_ = *g*_*zz*_*µ*_B_*B*_*z*_ ≈ 0.11 cm^−1^. At this field, no optical phonon (starting at ≈50–60 cm^−1^) is resonant with the KD gap and thus able to induce direct relaxation. We note that although extending phonon simulations to the full Brillouin zone would allow us to estimate the direct relaxation rate, this will not match the experimental values of relaxation as they are likely dominated by dipolar effects. This picture is further supported by a recent, independent first-principles study of the direct process in magnetically diluted Ln-based coordination polymers,^28^ which shows that an appreciable direct-process contribution to relaxation only emerges above 3000 Oe, *i.e.,* an order of magnitude larger than both the field used here to lift the Kramers degeneracy and the experimental field of Bode *et al.* We therefore conclude that a single-phonon direct process alone cannot be responsible for the observed *T*_1_ relaxation of the Yb(tren-x) systems in the field and temperature regime considered here, and we postpone addressing the ultra-low-*T* spin dynamics to future work.

As expected from the magnitude of the crystal-field gaps, Orbach relaxation is strongly suppressed at low temperatures, leaving Raman relaxation as the dominant mechanism. The simulated relaxation times are consistent with experimental data over the temperature range where Raman relaxation prevails, while additional mechanisms become relevant below a few degrees Kelvin, such as quantum tunnelling of the magnetization,^29,30^ or dipole–dipole interactions between electronic spins. In our calculations, we assume perfectly diluted crystals of Yb(tren-) but in practice, even at 1% Yb concentration, such interactions can affect the relaxation dynamics at low temperatures, reducing *T*_1_ and contributing to deviations from the simulated values. A comprehensive set of measurements at higher temperatures would therefore be highly valuable to further assess the agreement between our model and the experimental data. Nonetheless, our energy cut-off analysis shows that for *T* ∼ 10 K, only phonons within a low-energy window of ∼60 cm^−1^ are relevant. At first glance, the minor structural differences among the variants, together with their nearly identical electronic spectra, would suggest an essentially unchanged low-energy spin–phonon coupling. Indeed, the structural perturbations are highly localized and spatially distant from the Yb ions, and would therefore be expected to primarily affect high-energy vibrational modes. Contrary to this expectation, Fig. 4 reveals marked differences in *D*(*ω*) already in the low-energy regime. These results demonstrate that even minimal structural changes can significantly modify low-energy spin–phonon coupling, leading to non-trivial variations in relaxation rates that originate from collective changes across the low-energy phonon manifold rather than from individual vibrational modes. These findings highlight the high sensitivity of low-temperature spin dynamics to weak spin–phonon interactions and show that even subtle chemical modifications in the second coordination sphere provide an effective handle to tune relaxation pathways.

Importantly, these observations are at odds with the conventional approach to the chemical design of magnetic molecules. Until recently, the principal route toward the design of magnetic molecules has been the tailoring of crystal field excitations through the modification of the ions' first coordination shell. The overwhelming success of this strategy could potentially reinforce the idea that spin–phonon coupling could also be tackled this way, and attempts to control and rationalize Raman relaxation through tailoring of molecular structure have been made.^17,31,32^ However, the present results demonstrate that this conceptual approach to the chemical design of magnetic molecules is no longer applicable when considering the problem of tuning spin–phonon coupling. Different from the well understood correlation between an ion's crystal field and first-coordination shell modification, even localized chemical perturbations at the structural level result in delocalized and non-trivial modifications of vibrational spectra and spin–phonon couplings to a degree that it is not possible to establish a clear cause-and-effect relation. This state of affairs clearly represents a challenge for the entire community, with decades-long consolidated approaches to the synthesis of molecular magnets in need of an update. At the same time, we believe that these outstanding challenges also constitute an opportunity for further development. The establishment of a systematic understanding of THz vibrations and their relation to molecular and crystal structure would have a broad impact on many different branches of chemistry,^33^ physics and materials science as well as positive repercussions of technologies ranging from heat transport^34^ to vibrational spectroscopic imaging.^35^ A range of tools is already available to pursue this challenge. On the one hand, first-principles methods like the ones used in this work are nowadays capable of providing quantitative predictions of relaxation times across a wide range of temperatures and stand out as a mature tool to address the correlation between spin–phonon coupling and molecular structure. Recently, these tools have also been accelerated through machine learning surrogate models^36^ as well as integrated into molecular discovery frameworks,^37–39^ and further development in these directions holds transformative potential for the field of molecular magnetism. On the other hand, a range of scattering and spectroscopic techniques have recently been used to support theoretical explorations of spin–phonon coupling, *e.g.* neutron or X-ray scattering,^40–42^ far-infrared or Raman magnetospectroscopy,^6,43,44^ nuclear resonance vibrational spectroscopy,^45^ and THz electron paramagnetic resonance.^46^ These techniques are increasingly successful in probing the spin–phonon coupling of phonons in resonance to spin transitions, and the exploration of their remit in sensing those low-energy phonons leading to virtual transitions and Raman relaxation appears to be a very promising possible future direction for the field to take.

## Conclusions

In conclusion, we have here used three Yb(iii) compounds to isolate the correlation between molecular structure modifications and spin–phonon coupling and relaxation, going beyond well-documented first-coordination-shell effects on crystal field excitations. The absence of a simple chemical interpretation of our quantitative simulations highlights the urgency to extend the existing frameworks for the molecular design of magnetic molecules beyond simple magneto-structural correlations and towards building a mapping between molecular structure and delocalized THz phonons and corresponding spin–phonon couplings. We anticipate that further development and integration of chemical synthesis, spectroscopic methods, and predictive first-principles frameworks hold the key to solving this challenge.

## Computational methods

The cell and geometry optimization process and the simulations of *Γ*-point phonons for the unit cells of 1, 2 and 3 were performed through periodic density functional theory (pDFT) calculations using the CP2K package.^47^ The original X-ray crystallographic structure was used as a starting point. Cell optimization was performed employing a tight convergence criterion of 10^−7^ a.u. for the forces and a Self Consistent Field (SCF) convergence criterion of 10^−10^ a.u. for the energy. A plane wave cutoff of 1000 Ry, DZVP-MOLOPT Gaussian basis sets, and Goedecker–Teter–Hutter pseudopotentials^48^ were employed for all atoms. The Perdew–Burke–Ernzerhof (PBE)^49^ functional was used, together with Grimme's D3 dispersion correction to properly describe van der Waals interactions in the molecular crystal. Phonon modes *Q*_*α*_ and the corresponding frequencies *ω*_*α*_ were computed with a two-step numerical differentiation of forces and a step of 0.01 Å. By means of the ORCA (v. 5) quantum chemistry package,^50^ electronic structure and magnetic properties were computed on the optimized structures. The DKH-def2-TZVPP basis set was used for all the atoms, except Yb, for which SARC2-DKH-QZVP was employed. Douglas–Kroll–Hess (DKH) scalar relativistic correction to the electronic Hamiltonian was employed.^51,52^ Picture-change effects were included in the calculation. Multireference calculations were performed using Complete Active Space Self Consistent Field (CASSCF). The active space used to build the CASSCF wavefunction is (13, 7), *i.e.*, thirteen electrons in seven 4f-orbitals. The n-electron valence state perturbation theory at second order (NEVPT2 (ref. 53)) was applied to include corrections on the three systems. The spin–phonon coupling coefficients (∂*B*^*l*^_*m*_/∂*Q*_*α*_) were calculated by resorting to two-step numerical differentiation of the crystal field parameters *B*^*l*^_*m*_ using a step of 0.01 Å for each molecular degree of freedom. Their value was then retrieved through the interpolation with a second-order polynomial function, keeping the resulting linear coefficients. The Zeeman term for the static Hamiltonian in 2 was built assuming an external static magnetic field of 0.05 T, oriented along the **ẑ** direction of the external Cartesian reference frame. A rotation to align the magnetic field with the terms of the Hamiltonian expressed in the molecular reference frame was also applied by specifying Euler's angles that define the rotation. Dirac's delta functions appearing in the rate equations (see eqn (8) and (11)) were smeared out with a Gaussian function with *σ* = 10 cm^−1^. Second and fourth-order time-local quantum master equations (eqn (6) and (9)), were used to simulate one- and two-phonon relaxation processes, respectively. In practice, *T*_1_ is extracted from the matrix *R*_*ba*_ of eqn (12) as the first non-null eigenvalue. The software MolForge was used for these simulations.^23,54–56^

## Author contributions

Conceptualization: A. L., P. S., and S. C.; data curation: G. S. and L. A. M.; formal analysis: G. S. and L. A. M.; funding acquisition: A. L. and S. C.; investigation: G. S. and L. A. M.; methodology: G. S., L. A. M., and A. L.; project administration: A. L.; resources: A. L. and S. C.; software: G. S., L. A. M., and A. L.; supervision: A. L., P. S., and S. C.; validation: G. S. and L. A. M.; visualization: G. S., L. A. M., and P. S.; writing – original draft: G. S.; writing – review & editing: G. S., L. A. M., A. L., P. S., and S. C.

## Conflicts of interest

There are no conflicts to declare.

## Supplementary Material

SC-017-D6SC02836E-s001

SC-017-D6SC02836E-s002

## Data Availability

All the spin dynamics simulations discussed in the present manuscript have been carried out with the software MolForge, which can be obtained at https://github.com/LunghiGroup/MolForge. All data needed to evaluate the conclusions in the paper are present in the paper and/or the supplementary information (SI). Additional data related to this paper may be requested from the authors. Supplementary information is available. See DOI: https://doi.org/10.1039/d6sc02836e.
