# Supplementary material for: Raman relaxation in Yb(iii) molecular qubits: non-trivial correlations between spin–phonon coupling and molecular structure
Source: Chem Sci. 2026 Sep 14. Online ahead of print. doi: 10.1039/d6sc02836e (PMC13618585; doi:10.1039/d6sc02836e)
Supplement: Supplementary file 1 [file SC-OLF-D6SC02836E-s001.pdf]

Supplementary Information (SI) for Chemical Science.
This journal is © The Royal Society of Chemistry 2026
Supplementary Information (SI) for Chemical Science.
This journal is © The Royal Society of Chemistry

Supplementary materials for
Raman relaxation in Yb(III) molecular qubits: non-trivial correlations between spin-phonon coupling and molecular structure

Giacomo Sansone
Università di Parma, Dipartimento di Scienze Matematiche, Fisiche e Informatiche, I-43124 Parma, Italy and INFN–Sezione di Milano-Bicocca, gruppo collegato di Parma, 43124 Parma, Italy

Lorenzo A. Mariano and Alessandro Lunghi*
School of Physics, AMBER and CRANN Institute, Trinity College, Dublin 2, Ireland

Stefano Carretta and Paolo Santini
Università di Parma, Dipartimento di Scienze Matematiche, Fisiche e Informatiche, I-43124 Parma, Italy INFN–Sezione di Milano-Bicocca, gruppo collegato di Parma, 43124 Parma, Italy and Consorzio Interuniversitario Nazionale per la Scienza e Tecnologia dei Materiali (INSTM), I-50121 Firenze, Italy

I. Γ-POINT CALCULATIONS

In principle, a full Brillouin zone integration would be desirable to fully capture the phonon density of states relevant to spin relaxation. However, some of us have addressed this point previously and concluded that a full Brillouin Zone integration has minimal effects on computed rates and that the vanishingly small effect of this additional calculation is not worth its extremely large computational overheads.

Regarding the cost, taking the Yb(trensal) as a representative example, a single unit cell of this system contains approximately 250 atoms. Therefore, even the smallest possible calculation needed to estimated phonons' dispersions would require the DFT optimization of a simulation cell of about 2000 atoms (2x2x2 supercell) plus the subsequent simulation of 1,500 single-point force calculations for the computation of the Hessian matrix. Whilst possible in principle, this is an enormous computational overhead.

Most importantly, we argue that the benefit introduced by this extensive number of additional

* lunghia@tcd.ie

calculations would very likely have minimal, if any, impact on the computed rates. A previous work of one of the co-authors on a molecular crystal of a Co^{2+} single-ion magnet (see J. Chem. Phys. 153, 174113, 2020), explicitly benchmarked relaxation times computed at the Γ-point against fully converged calculations integrated over the Brillouin zone for a real molecular spin system. There, both Orbach and Raman relaxation times estimated at the Γ-point were found in close agreement with those obtained from dense meshes (up to 8^3 and 4^3 q-points, respectively) across the whole temperature range investigated. This result has a simple physical explanation: optical phonons, exhibiting small dispersions, drive both Orbach and Raman relaxation instead of acoustic ones, making a slightly smeared Γ-point calculation virtually indistinguishable from a fully converged Brillouin Zone integration.

We further note that the Gaussian smearing width adopted in our simulations ($\sigma = 10$ cm^{-1}, chosen after a convergence check as shown in Figure S1) is comparable to or larger than the smearing found in the above-mentioned benchmark to reproduce fully converged, Brillouin-zone-integrated relaxation times at the Γ-point, providing an additional, quantitative safeguard against the finite-size effects raised by the reviewer.

We believe that the arguments from the literature presented above provide solid support for the validity of the Γ-point approach adopted in our study.

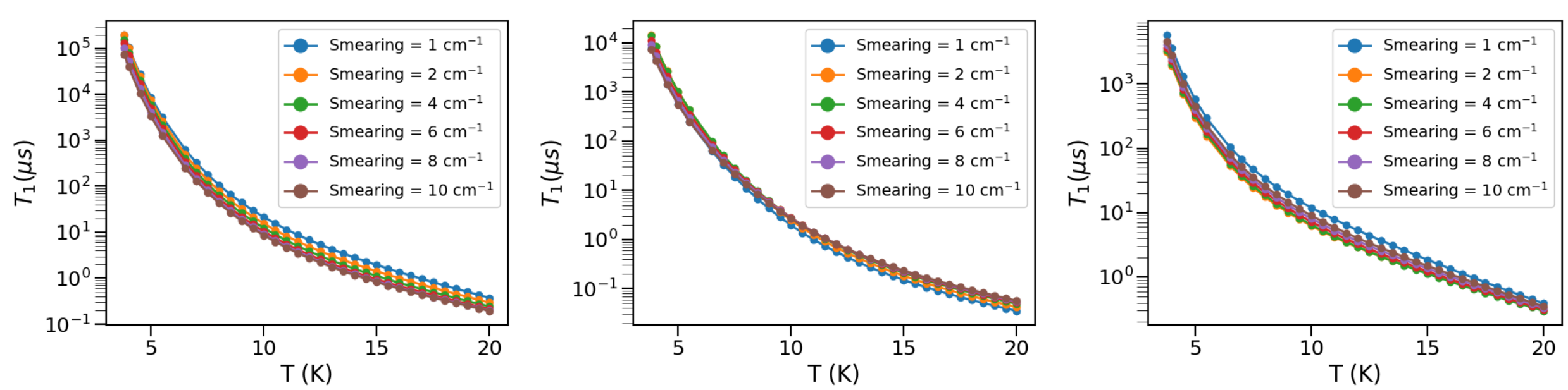

Figure S1: Convergence check of the relaxation curves upon variation of the Gaussian smearing parameter σ. **Left**: Yb(trensal), **Center**: Yb(trenpvan), **Right**: Yb(trenovan).

II. THE IMPACT OF AXIAL SYMMETRY BREAKING ON STATIC AND DYNAMIC PROPERTIES

The non-axially symmetric g-tensor obtained from first-principles calculations is in contrast with the experimental evidence, where the C_3 symmetry is preserved for the three systems. However,

the proposed solution of forcing molecular symmetry constraints when performing the structure optimization of the unit cell is not viable in the present context as it would lead to imaginary low-energy phonon frequencies, invalidating the whole procedure.

To get a deeper insight into the impact that symmetry has on the static and dynamic properties of these systems, we took a slightly different route. Firstly, we symmetrized the single-molecule structure of Yb(trensal) and Yb(trenovan), enforcing the C_3 symmetry, and performed a CASSCF calculation on the new geometries. We show the energy spectra and the g-tensors of the two molecules in Tables S1 and S2, respectively. As can be seen, this procedure allows the retrieval of the axial symmetry of the g-tensors. However, there is no substantial impact on the KD energies, which remain approximately unaltered with respect to the ones obtained with the original simulations.

A further step was then made by evaluating the impact of symmetry on the relaxation dynamics. We started by considering the spin-phonon couplings for the atoms belonging to the sole first coordination sphere of Yb (i.e. 4 nitrogen and 3 oxygen atoms). Then, resorting to the same procedure described in the Methods section of the main file, we re-computed the relaxation dynamics as a function of temperature for both Raman and Orbach processes by means of the Molforge software. In the left panels of Figures S2 and S3 (also added to SI), we compare the results of these simulations with the data presented in the main article and obtained by considering the spin-phonon couplings of all the atoms belonging to the molecule. This test allows us to prove that the dynamics is most entirely dominated by the interactions involving first-sphere atoms. Thanks to this, we moved on to computing spin-phonon coupling for the first coordination shell of the symmetrized molecules (without changing the phonons computed at the non-symmetrized molecular geometry). The results, for both Raman and Orbach processes, are shown in the right panels of Figures S2 and S3 for Yb(trensal) and Yb(trenovan), respectively. As can be inferred from these plots, the axial symmetry breaking originated from the unit cell optimization leads to a vanishingly small change in the relaxation dynamics. All these observations allow us to support the idea that lowering the symmetry of the molecules by performing DFT energy optimization does not lead to a significant change in the results, both from a qualitative and quantitative viewpoint.

A. Energy spectra

Molecule	Method	KD1 (cm^{-1})	KD2 (cm^{-1})	KD3 (cm^{-1})	KD4 (cm^{-1})
Yb(tren**sal**)	Sim.	0.0	430.7	784.2	912.7
	Sim. (symmetrized)	0.0	428.3	788.4	910.3
	Exp.	0.0	464	737	913
Yb(tren**ovan**)	Sim.	0.0	378.3	776.8	909.8
	Sim. (symmetrized)	0.0	379.0	778.8	914.3
	Exp.	0.0	479	819	980

Table S1: Comparison between simulated (without and with symmetrization) and experimental electronic spectra for the ground term of Yb(trensal) and Yb(trenovan).

B. *g*-tensor

Molecule	Method	g_{xx}	g_{yy}	g_{zz}
Yb(tren**sal**)	Sim.	2.68	2.73	4.88
	Sim. (symmetrized)	2.75	2.76	4.76
	Exp.	2.9	2.9	4.3
Yb(tren**ovan**)	Sim.	2.46	2.61	5.24
	Sim. (symmetrized)	2.61	2.61	5.07
	Exp.	2.65	2.65	4.89

Table S2: Comparison between simulated (without and with symmetrization) and experimental g-tensor values for Yb(trensal) and Yb(trenovan).

C. Effects on dynamics

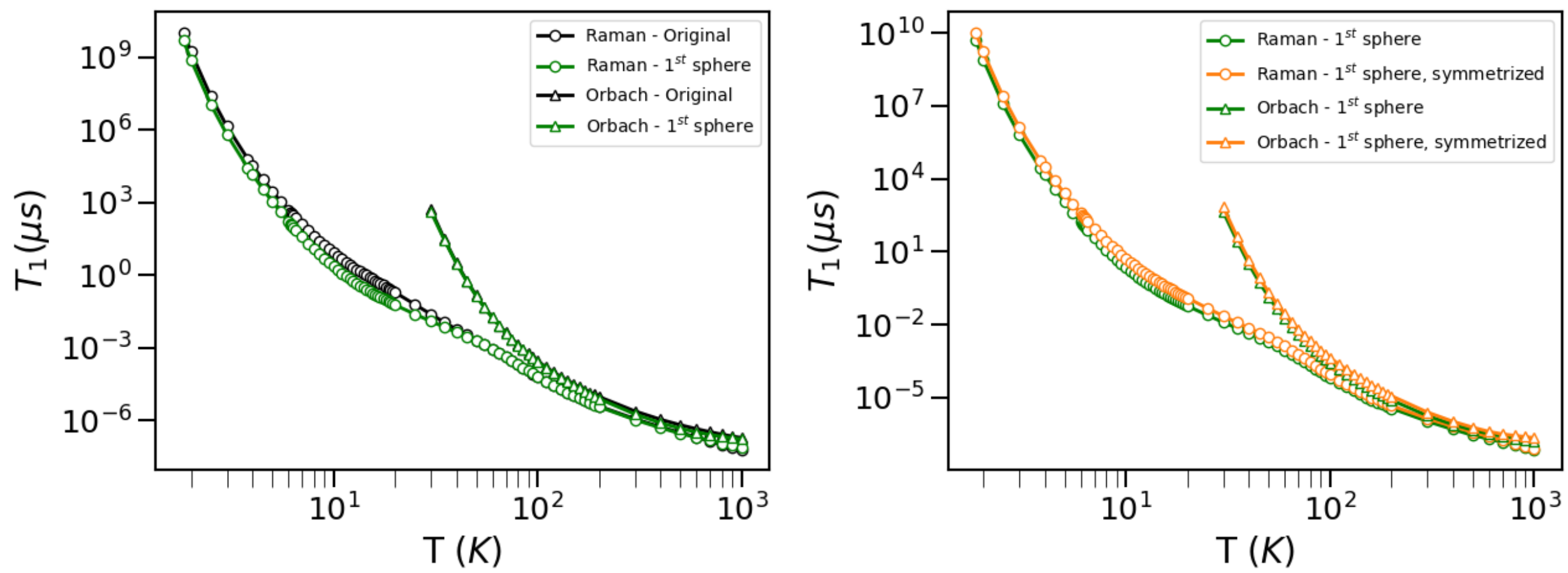

Figure S2: **Yb(trensal)**. **Left panel**: Comparison between the relaxation dynamics originated by the spin-phonon couplings of all the atoms in the molecule and the one obtained by restriction within the first coordination sphere of the Yb atom (4 Nitrogen atoms, 3 Carbon atoms). **Right panel**: relaxation dynamics induced by Yb first coordination sphere in the original molecule (optimized through DFT) and the symmetrized one.

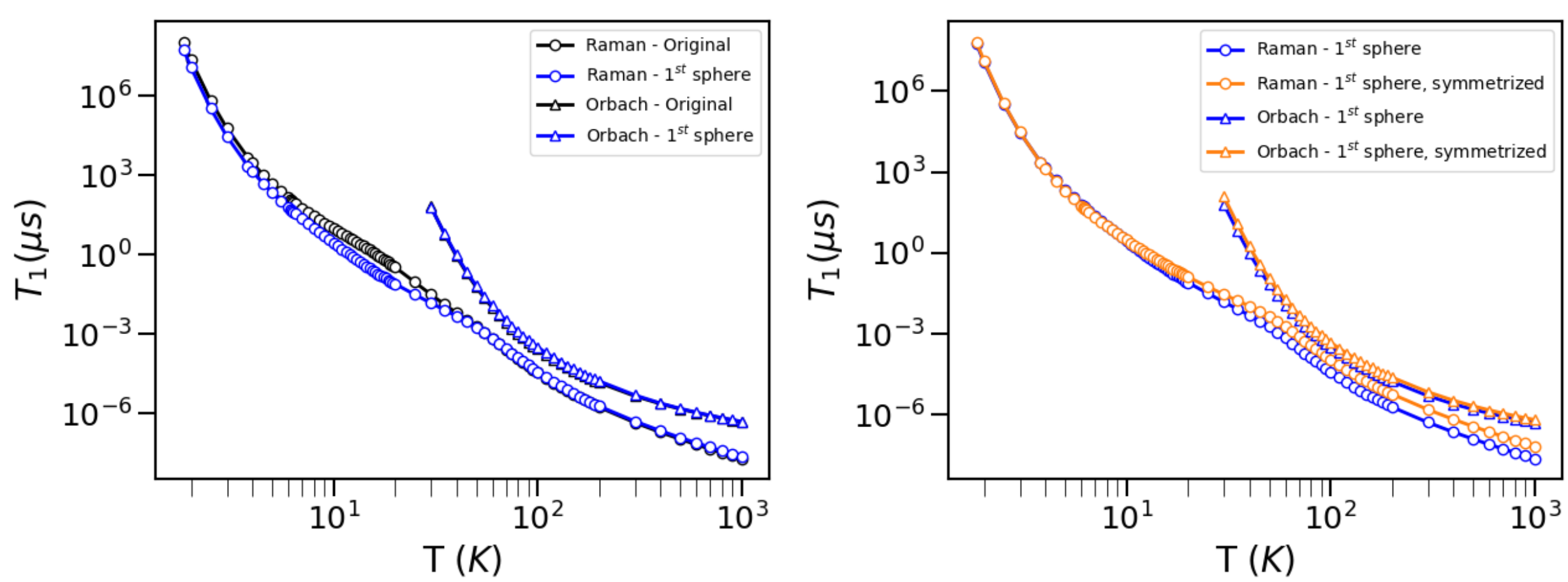

Figure S3: **Yb(trenovan)**. **Left panel**: Comparison between the relaxation dynamics originated by the spin-phonon couplings of all the atoms in the molecule and the one obtained by restriction within the first coordination sphere of the Yb atom (4 Nitrogen atoms, 3 Carbon atoms). **Right panel**: relaxation dynamics induced by Yb first coordination sphere in the original molecule (optimized through DFT) and the symmetrized one.

III. MAGNETIC FIELD DEPENDENCE

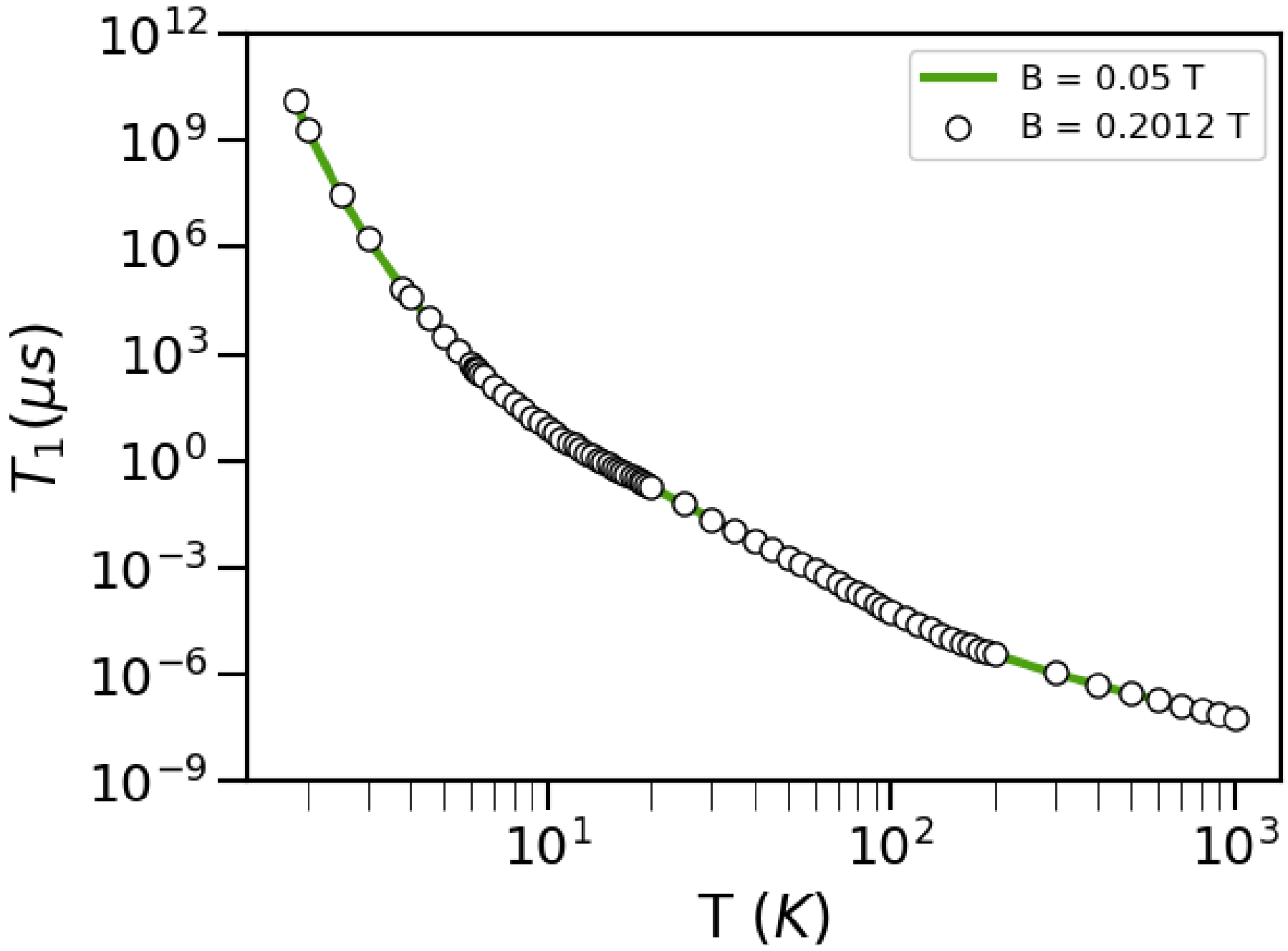

Figure S4: Relaxation time T_1 as a function of temperature for two different values of the external magnetic field (the spatial orientation of the field is the same).

IV. RELAXATION DYNAMICS

A. Raman vs Orbach relaxation

Here, we show the relaxation dynamics of the three systems for a broader range of temperatures, ranging from 2 to 1000 K, in order to facilitate the comparison at high temperatures between Raman- and Orbach-induced relaxation. Although we lack of experimental information above 10 K, theoretical predictions suggest Raman processes to be the dominant source of T_1 decay over the Orbach ones.

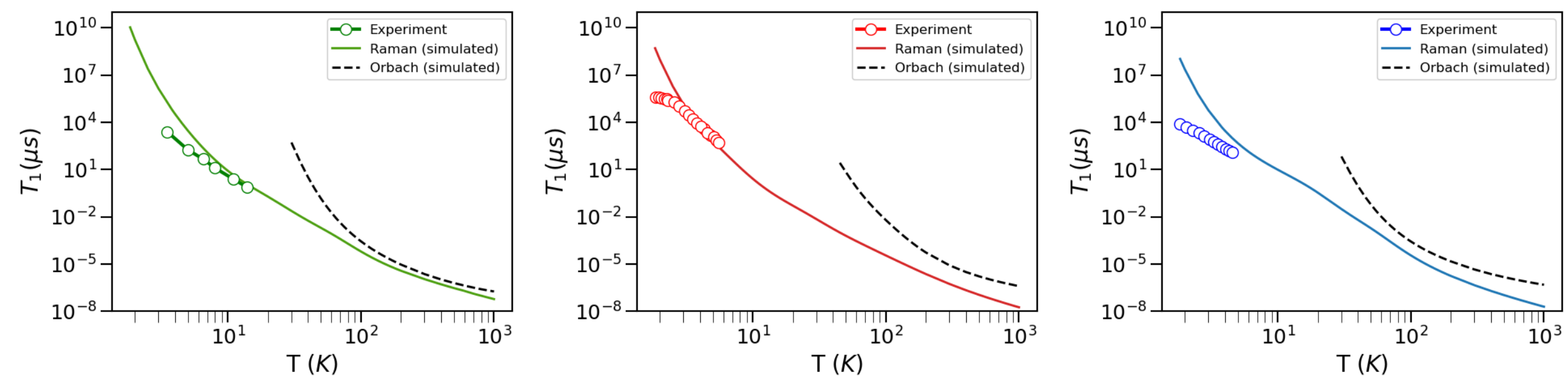

Figure S5: Relaxation dynamics of the three systems for a broader range of temperatures. Left: Yb(trensal). Center: Yb(trenpvan). Right: Yb(trenovan).

B. Direct comparison within the Yb(tren-x) family

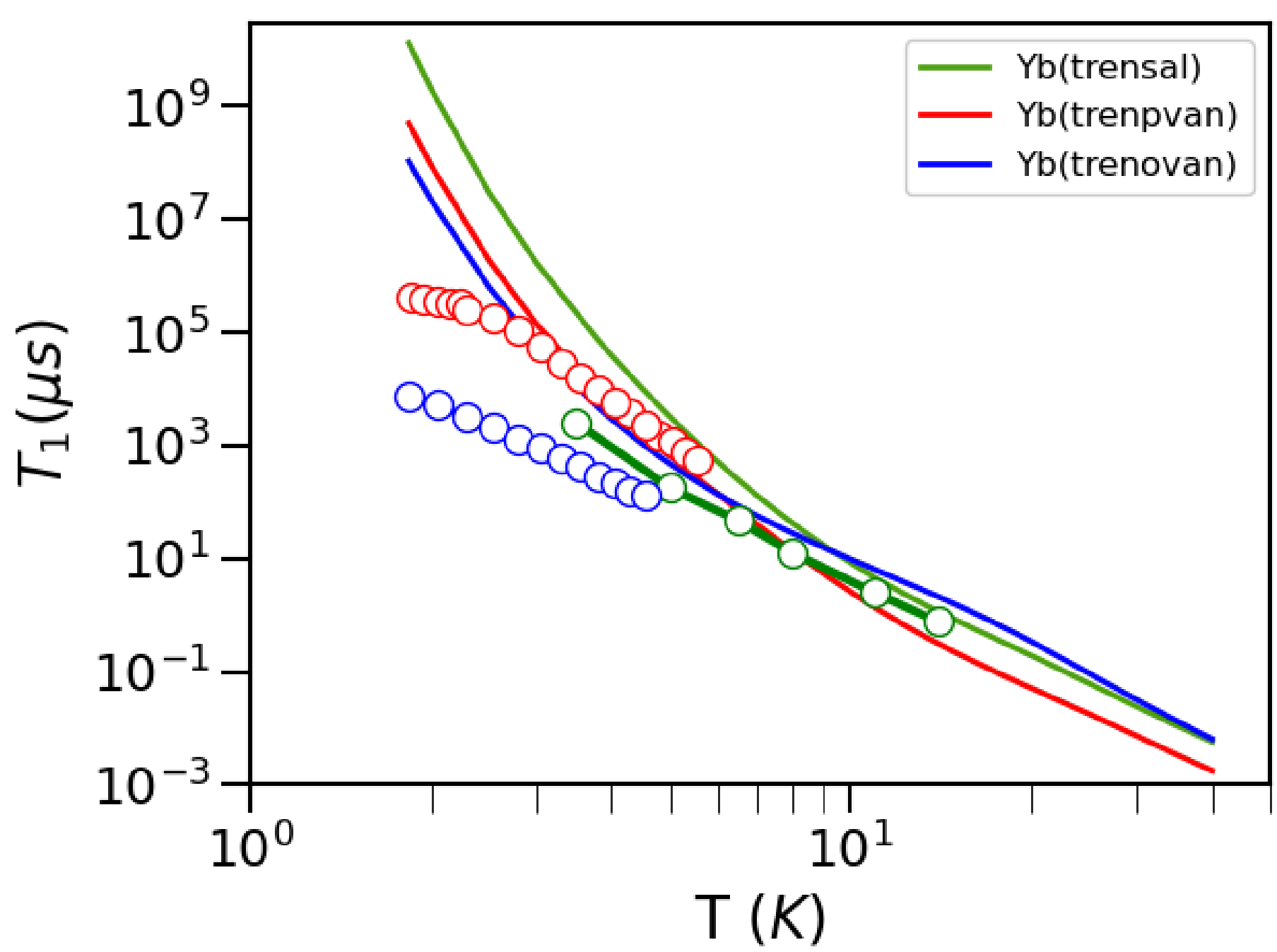

Figure S6: Direct comparison of the relaxation dynamics of the three systems in the temperature range between 1 and 50 K. Continuous line are the simulated curves, whereas hollow circles are the experimental data.

C. The role of crystal field in relaxation

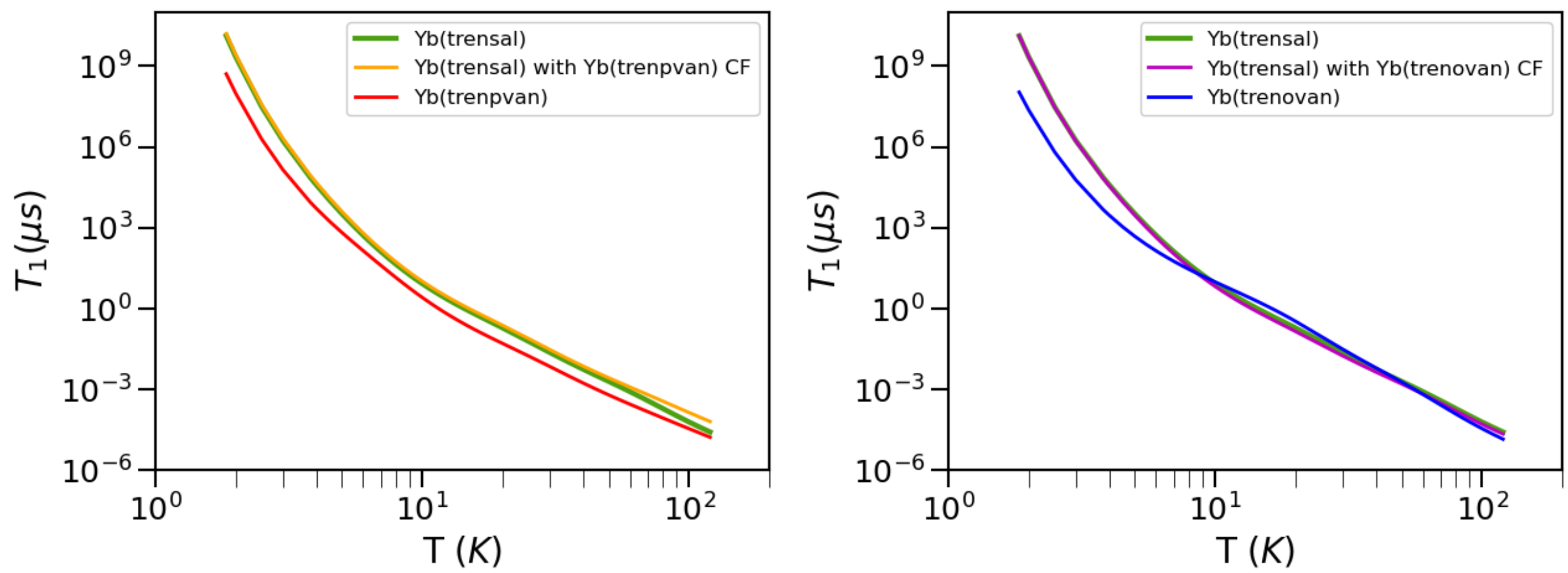

Figure S7: Relaxation dynamics of Yb(trensal) compared to the one of Yb(trensal) itself after interchanging its static crystal field Hamiltonian with the one of Yb(trenpvan) and Yb(trenovan).

V. INFRA-RED SPECTRA

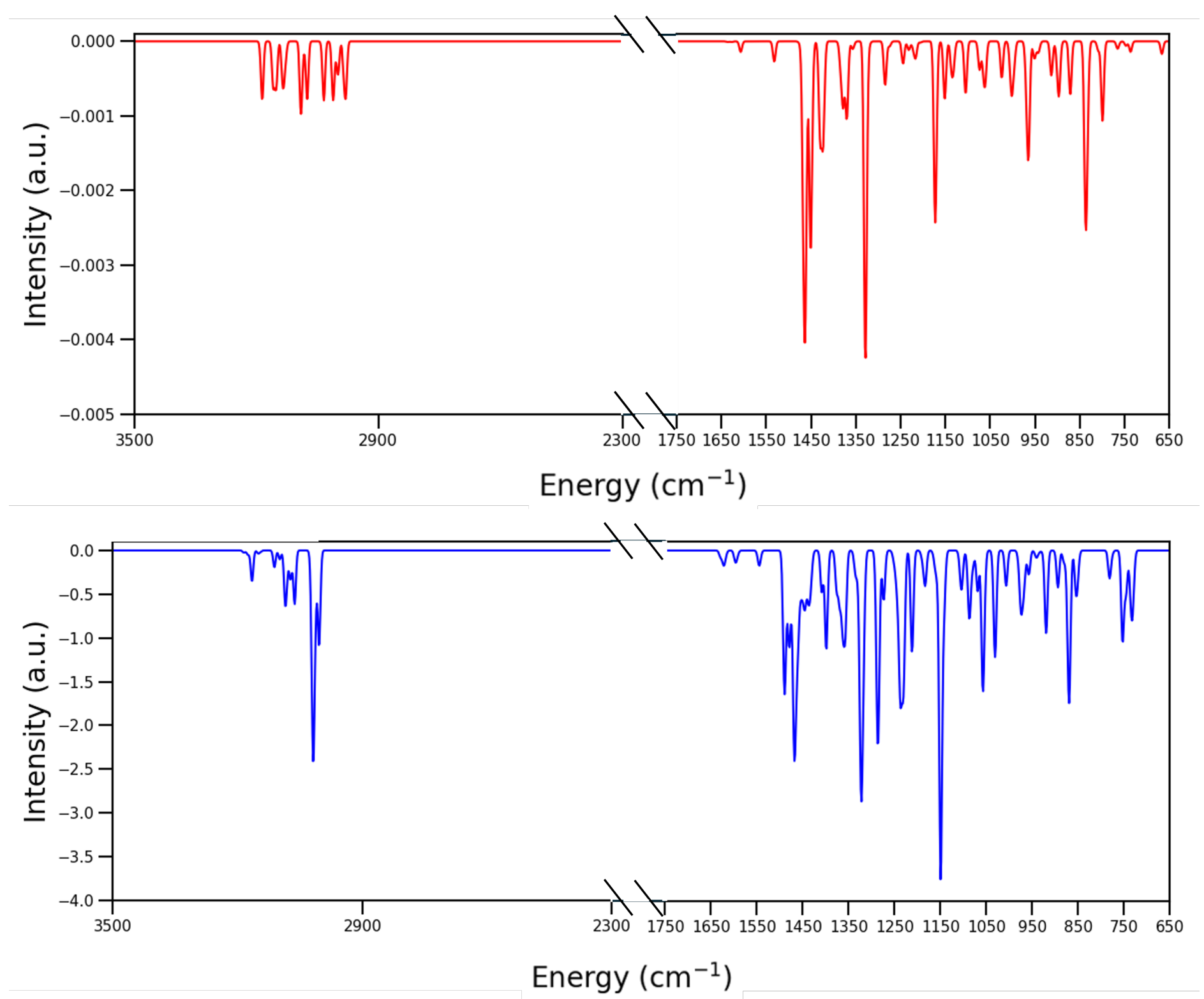

Figure S8: Infra-red spectra for Yb(trenpvan) (top) and Yb(trenovan) (bottom).
